# Effects of Various Temperatures and pH Values on the Extraction Yield of Phenolics from Litchi Fruit Pericarp Tissue and the Antioxidant Activity of the Extracted Anthocyanins

**DOI:** 10.3390/ijms9071333

**Published:** 2008-07-22

**Authors:** Neungnapa Ruenroengklin, Jia Zhong, Xuewu Duan, Bao Yang, Jianrong Li, Yueming Jiang

**Affiliations:** 1 South China Botanical Garden, Chinese Academy of Sciences, Guangzhou 510650, P.R. China; 2 College of Food Science and Biotechnology and Environmental Engineering, Zhejiang Gongshang University, Hangzhou, 310035, P.R. China

**Keywords:** Litchi, extraction, temperature, pH, phenolics, anthocyanin, antioxidant activity

## Abstract

Litchi fruit pericarp tissue is considered an important source of dietary phenolics. This study consisted of two experiments. The first was conducted to examine the effects of various extraction temperatures (30, 40, 50, 60, 70 and 80 °C) and pH values (2, 3, 4, 5 and 6) on the extraction yield of phenolics from litchi fruit pericarp. Extraction was most efficient at pH 4.0, while an extraction temperature of 60 °C was the best in terms of the combined extraction yield of phenolics and the stability of the extracted litchi anthocyanins. The second experiment was carried out to further evaluate the effects of various temperatures (25, 35, 45, 55 and 65 °C) and pH values (1, 3, 5 and 7) on the total antioxidant ability and scavenging activities of DPPH radicals, hydroxyl radical and superoxide anion of the extracted anthocyanins. The results indicated that use of 45–60 °C or pH 3–4 exhibited a relatively high antioxidant activity. The study will help improve extraction yield of phenolics from litchi fruit pericarp and promote better utilization of the extracted litchi anthocyanins as antioxidants.

## 1. Introduction

Litchi (*Litchi chinensis Sonn*.) is a subtropical fruit that originated in South-East Asia [1]. In recent years, litchi production has increased steadily around the world. Litchi fruit pericarp (LFP) accounts for approximately 15% by weight of the whole fresh fruit [2] and contains significant amounts of phenolics, among which anthocyanins are the major polyphenols. Anthocyanins play an important pharmacological role against various human diseases, such as cardiovascular disease, cancer, inflammation and allergies [3–6]. Furthermore, some studies have indicated that LFP is a powerful free radical scavenger and exhibits strong antioxidant activity [7, 8], which suggest its use as a readily accessible source of natural antioxidants and/or a possible supplement in the food or pharmaceutical industry [2, 6].

Extraction yields of phenolics from plant tissues depend on the various extraction conditions [2]. Most phenolics present in plant tissues are soluble in polar solvents and can be extracted using methanol containing small amount of hydrochloric or formic acid [3, 9, 10]. The low pH value of the extraction solution can prevent the oxidation of phenolics, while the use of low temperatures may preserve anthocyanin stability [2, 11]. Thus, an investigation of the efficient extraction of phenolics from LFP requires evaluation under various temperatures and solvent pH values.

Anthocyanins are the major phenolics present in LFP. The major anthocyanins from LFP tissues were identified as epicatechin, proanthocyanidin B4 and proanthocyanidin B2 [2, 10]. Anthocyanins show good antioxidant ability [7], but they are relatively unstable [9]. As the stability of litchi anthocyanins is dependent on various factors, such as pH value and temperature [12], the antioxidant ability of the litchi anthocyanins under the conditions of various temperatures and pH values also needs further evaluation.

The objective of this study was to examine the effects of various extraction temperatures and pH values on the extraction yield of phenolics from LFP and then evaluate total antioxidant ability and scavenging activities towards α,α-diphenyl-β-picrylhydrazy (DPPH) and hydroxyl radicals and superoxide anion of the extracted litchi anthocyanins under various temperatures and pH conditions. The study will help improve extraction of phenolics from litchi fruit pericarp and promote better utilization of the extracted litchi anthocyanins as antioxidants.

## 2. Results and Discussion

**Figure 1.**
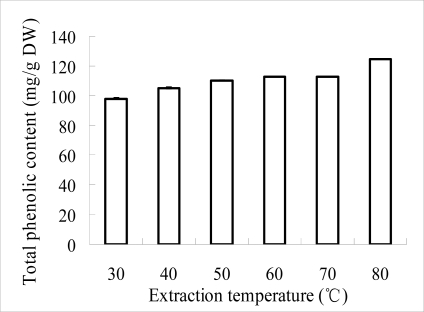
The effects of various temperatures on extraction yield of phenolics from pericarp tissues of litchi fruit.

**Figure 2.**
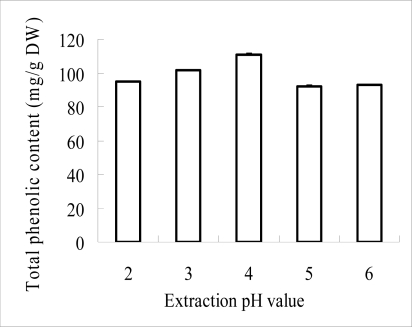
The effects of various pH values on extraction yield of phenolics from pericarp tissues of litchi fruit.

The extraction yield of phenolics from litchi fruit pericarp increased as the extraction temperature increased betwenn 30–80 °C (Figure 1), which suggested that litchi phenolics are relatively stable under high temperature conditions. Significant differences existed among 30, 40, 50 and 60 °C but did not appear between 60 and 70 °C. Thus, in practice an extraction temperature of 60 °C could be used, basedon the combined effects of the good extraction yield of phenolics and the stability of litchi anthocyanins [12]. As shown in Figure 2, the extraction yield of phenolics from LFP increased as the pH values increased from 2 to 4, but it decreased as the pH values higher than 4 were used, which indicated that the extraction pH value significantly affected the extraction yield compared with other factors. The increased extraction yield from LFP under the low pH conditions could be due to the inhibition of the enzymatic oxidation of phenolics and/or the maintenance of the extracted anthyocyanin stability [2, 12]. As the extraction efficiency and anthocyanin stability depend largely on the combined effects caused by temperature and pH of extraction solution [11], further investigation to optimize the two extraction factors could be required.

**Figure 3.**
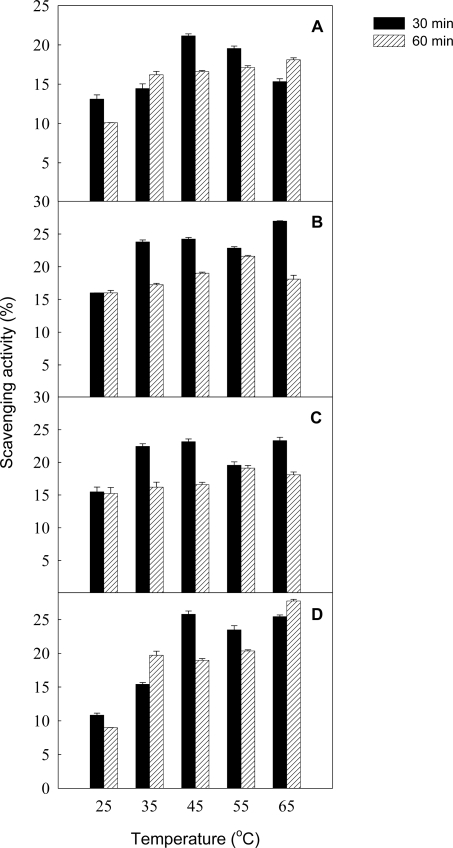
The effects of various temperatures on total antioxidant ability (A) and scavenging activities of DPPH radical (B), hydroxyl radical (C) and superoxide anion (D) of litchi anthocyanin.

Litchi anthocyanins from fruit pericarp tissues were readily purified by Amberlite XAD-7 chromatography column [10], which gave a good purification of anthocyanins from plant tissues and had no effect on the anthocyanin compositions [13]. In this study, the partially purified litchi anthocyanins were used to further examine the effect of various temperatures and pH values on the antioxidant activity. As shown in Figure 3, the antioxidant activity of the litchi anthocyanins was enhanced with increasing temperature up to 45 °C, but it decreased as the incubation time was extended to 60 min. Furthermore, the antioxidant ability of litchi anthocyanins for 30 min at 45 °C was significantly higher than that 55 °C. This study suggested that an appropriate temperature maintained a high antioxidant activity of litchi anthocyanins, which could be due to the combined effect of non-enzymatic reaction and anthocyanin stability [14].

**Figure 4.**
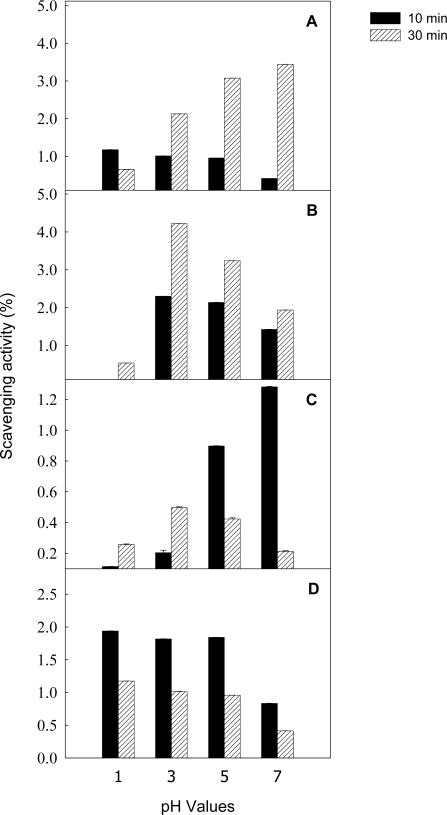
The effects of various pH values on total antioxidant ability (A) and scavenging activities of DPPH radical (B), hydroxyl radical (C) and superoxide anion (D) of litchi anthocyanin.

Figure 4 shows the effects of various pH values on total antioxidant ability and scavenging activities of litchi anthocyanins against DPPH and hydroxyl radical and superoxide anion. The superoxide anion scavenging activity of litchi anthocyanins was relatively stable at pH 1–5. However, there was a higher scavenging DPPH radical activity at pH 3–5, which was significant compared with pH 1 or 7. In addition, the total antioxidant ability of litchi anthocyanins was enhanced as the incubation time was extended from 30 to 60 min. Thus, pH values in the reaction medium significantly influenced the total antioxidant ability and scavenging activities of litchi anthocyanins against DPPH radicals, hydroxyl radical and superoxide anion, which could be due to the anthocyanin stability [12].

## 3. Conclusions

Various temperatures and pH significantly influenced the extraction yield of phenolics from LFP and the antioxidant activity of the extracted litchi anthocyanins. Application of pH 4.0 exhibited the most efficient extraction while the extraction temperature of 60 °C could be used in terms of the combined effects of the extraction yield of phenolics and the stability of the extracted litchi anthocyanin. Furthermore, the temperatures from 45–60 °C and pH values from 3 to 4 exhibited a relatively high antioxidant activity. As the extraction efficiency and the antioxidant activity of the extracted litchi anthocyanins depend largely on the combined effects of temperature and pH [11,14], further investigation to evaluate the two extraction factors by use of the artificial neural network could be required.

## 4. Experimental Section

### 4.1. Plant materials

Fresh fruits of litchi (*Litchi chinensis* Sonn.) cv. Feizixiao were obtained from a commercial market in Guangzhou. Fruits were selected for uniformity and size and bruised or diseased fruits were then discarded. LFP tissues were collected, then dried and finally stored at −18 °C for the following experiments.

#### 4.2. Experiment 1: Effects of various temperatures and pH values on extraction yield of phenolics from LFP

In our previous experiments, the use of 60% ethanol for an extraction time of 3 h exhibited the highest phenolic content from LFP. Thus, the extraction conditions were used to examine the effects of various temperatures and pH values on the extraction yield in this study. The dried LFP samples (5 g) were extracted for 3 h by stirring with 60% (v/v) ethanol (100 mL) at 30, 40, 50, 60, 70 or 80 °C and pH 7, or pH 2, 3, 4, 5 or 6 adjusted by a diluted HCl solution and room temperature (25 °C). The homogenate was filtered through Whatman No. 1 filter paper under vacuum, collected, concentrated and then dried by a rotary evaporator under vacuum at 50 °C. The dried filtrate was re-dissolved in 60% (v/v) ethanol and then used for the analysis of total phenolic content. Total phenolic content was estimated with Folin-Ciocalteu reagent by the method of Singleton and Rossi [15], using gallic acid as a standard. Results were expressed as micrograms of gallic acid equivalents (GAE) per gram on dry weight (DW) basis.

#### 4.3. Experiment 2: Effects of temperatures and pH values on antioxidant activity of the extracted litchi anthocyanin

Litchi anthocyanin was extracted by the method of Zhang *et al*. [12]. The dried LFP tissues (50 g) were added to 1.5 M HCl solution in 95% ethanol (1 L) and then kept at 4 °C overnight. The extract solution was filtered through Whatman No. 1 paper and the filtrate was then collected as the crude litchi anthocyanin. The crude litchi anthocyanin was concentrated using a rotary evaporator under vacuum at 40 °C and purified by the method of Norbaek and Kondo [16]. The concentrated anthocyanin was loaded into Amberlite XAD-7 column (1.5 × 20 cm) and then eluted with acetonitrile-trifloroacetic acid-H_2_O (200 mL, 50:0.5:49.5, v/v/v) [17]. The largest anthocyanin fraction was collected and lyophilized. The lyophilized anthocyanin was dissolved in a small amount of methanol and then used to examine the effects of temperatures and pH values on the antioxidant activity. The anthocyanin content was determined using a Unic UV-2802 spectrophotometer at 535 nm. Litchi anthocyanin solution (1 mL) at 300 μg/mL was incubated at 25, 35, 45, 55 and 65 °C and pH 4 for 30 or 60 min in water baths, whereas the anthocyanin solution (0.1 ml) was mixed with 0.9 ml of 0.1 M Tris-HCl buffer solution at pH 1, 3, 5 or 7 and then incubated for 10 or 30 min at 25°C, prior to the measurements of total antioxidant ability and scavenging activities of DPPH radical, hydroxyl radical and superoxide anion. Aliquots of the mixture solution were used for the following measurements.

### 4.4. Total antioxidant activity

Linoleic acid solution was prepared according to the method of Surrey [18]. Total antioxidant activity of litchi anthocyanin was determined by the method of Orak [19]. The mixture solution (1 mL) mentioned above was added to linoleic acid solution (5.0 mL, 6 mg/mL in 99 % methanol) and then incubated for 10 min at 37 °C. A 0.1-mL aliquot of the mixture solution was added to 75% ethanol (4.7 mL), 0.1 M ammonium thiocyanate (0.1 mL) and 20 mM ferrous chloride (0.1 mL) in 3.5% HCl solution. The reaction was then allowed for 5 min at 30 °C in dark. The absorbance was measured at 500 nm. Total antioxidant activity was expressed as a percentage of lipid peroxidation value and calculated as the inhibition percentage = (OD_0_ − OD_1_) × 100 / OD_0_, where OD_0_ was the absorbance of the control sample without litchi anthocyanins and OD_1_ was the absorbance of the litchi anthocyanin sample.

### 4.5. Scavenging activity against DPPH radical

DPPH radical scavenging activity of litchi anthocyanins was evaluated by the method of Yamaguchi *et al*. [20]. Briefly, the mixture solution (0.1 mL) mentioned above was mixed with 0.1 M Tris-HCl buffer (0.5 mL, pH 7.4) and 0.1 mM DPPH in methanol (0.4 mL) solution. The reaction was allowed to proceed for 20 min at 30 °C in dark. Distilled water instead of litchi anthocyanin solution was used as the control. The scavenging activity of litchi anthocyanins against DPPH radicals was expressed as the inhibition percentage = (OD_0_ − OD_1_) × 100 / OD_0_, where OD_0_ was the absorbance of the control while OD_1_ was the absorbance of litchi anthocyanin sample.

### 4.6. Scavenging activity against hydroxyl radical

The assay of the hydroxyl radical scavenging activity of litchi anthocyanins was conducted in the Fe^2+^-EDTA-H_2_O_2_-deoxyribose systems [21]. Briefly, the mixture solution (0.1 mL) mentioned above was mixed with the reaction solution (0.9 mL) containing 0.1 mM Fe(SO_4_), 0.1 mM EDTA and 1.75 mM 2-deoxyribose in 0.2 M phosphate buffer (pH 7.4). Then, 1.0 mM ascorbic acid (0.1 mL) and 0.01 M H_2_O_2_ (0.1 mL) were respectively added to the reaction mixture and incubated for 10 min at 37 °C. Finally, 1% thiobarbituric acid (0.5 mL) and 2.8% tricholroacetic acid (1.0 mL) were added, mixed, and then boiled for 10 min. The absorbance was measured at 532 nm and the scavenging activity of hydroxyl radical was then calculated as the percentage against the deoxyribose degradation.

### 4.7. Scavenging activity against superoxide anion

Superoxide anion scavenging activity was assayed by the method of Duan *et al*. [7]. Briefly, the mixture solution (0.1 mL) mentioned above was added to the reaction solution (1 mL) containing 50 μM riboflavin, 200 μM EDTA, 0.5 mM nitro blue tetrazolium and 20 mM methionine in 0.1 M phosphate buffer (pH 7.4). The mixture solution was exposed to two 30 W fluorescent lamps for 20 min. The absorbance was measured at 560 nm and then calculated as the percentage of superoxide anion scavenging activity.

### 4.8. Statistical analysis

All analyses were performed in triplicate. Each value was the mean of three replicate determinations and the vertical bar indicated the standard error where it exceeded the symbol size. One-way analysis of variance (ANOVA) was carried out to test any significant difference between the means. Differences between the means at the 5% level were considered to be significant.
